# RETRACTED: Alhakamy et al. Fluoxetine Ecofriendly Nanoemulsion Enhances Wound Healing in Diabetic Rats: In Vivo Efficacy Assessment. *Pharmaceutics* 2022, *14*, 1133

**DOI:** 10.3390/pharmaceutics16020157

**Published:** 2024-01-23

**Authors:** Nabil A. Alhakamy, Giuseppe Caruso, Anna Privitera, Osama A. A. Ahmed, Usama A. Fahmy, Shadab Md, Gamal A. Mohamed, Sabrin R. M. Ibrahim, Basma G. Eid, Ashraf B. Abdel-Naim, Filippo Caraci

**Affiliations:** 1 Department of Pharmaceutics, Faculty of Pharmacy, King Abdulaziz University, Jeddah 21589, Saudi Arabia; nalhakamy@kau.edu.sa (N.A.A.); oaahmed@kau.edu.sa (O.A.A.A.); uahmedkauedu.sa@kau.edu.sa (U.A.F.); shaque@kau.edu.sa (S.M.); 2 Advanced Drug Delivery Research Group, Faculty of Pharmacy, King Abdulaziz University, Jeddah 21589, Saudi Arabia; 3 Center of Excellence for Drug Research and Pharmaceutical Industries, King Abdulaziz University, Jeddah 21589, Saudi Arabia; 4 Mohamed Saeed Tamer Chair for Pharmaceutical Industries, King Abdulaziz University, Jeddah 21589, Saudi Arabia; 5 Department of Drug and Health Sciences, University of Catania, 95125 Catania, Italy; annaprivitera01@gmail.com (A.P.); carafil@hotmail.com (F.C.); 6 Unit of Neuropharmacology and Translational Neurosciences, Oasi Research Institute—IRCCS, 94018 Troina, Italy; 7 Department of Natural Products and Alternative Medicine, Faculty of Pharmacy, King Abdulaziz University, Jeddah 21589, Saudi Arabia; gahussein@kau.edu.sa; 8 Preparatory Year Program, Batterjee Medical College, Jeddah 21442, Saudi Arabia; sabrin.ibrahim@bmc.edu.sa; 9 Department of Pharmacognosy, Faculty of Pharmacy, Assiut University, Assiut 71526, Egypt; 10 Department of Pharmacology and Toxicology, Faculty of Pharmacy, King Abdulaziz University, Jeddah 21589, Saudi Arabia; beid@kau.edu.sa (B.G.E.); aaabdulalrahman1@kau.edu.sa (A.B.A.-N.)

The journal retracts the article, “Fluoxetine Ecofriendly Nanoemulsion Enhances Wound Healing in Diabetic Rats: In Vivo Efficacy Assessment” [1]. 

Following publication, concerns were brought to the attention of the publisher regarding image reuse across several publications.

Adhering to our complaints procedure, an investigation was conducted that confirmed the duplication of Figure 5A in [1] with parts of Figure 1A of [2], Figure 2A of [3] and Figure 3A [4], presenting different experimental conditions.

While the authors fully cooperated with the Editorial Office during the investigation, they were unable to satisfactorily explain the overlap of figures and meet the required quality standards of raw images in order to consider a correction as per the journal’s original image requirements policy (https://www.mdpi.com/journal/pharmaceutics/instructions#oriimages). As a result, the Editorial Board and Editor-in-Chief were unable to confirm the reliability of the findings and subsequently decided to retract this paper.

This retraction was approved by the Editor-in-Chief of the journal *Pharmaceutics*. 

The authors did not agree to this retraction.
